# ZENIA: Development of an AI‐driven nutrition app to support immune health: An EAACI task force report

**DOI:** 10.1111/pai.70313

**Published:** 2026-02-27

**Authors:** Emilia Vassilopoulou, Beniam Burje, Gregorio Paolo Milani, Cevdet Ozdemir, Rosan Meyer, Berber Vlieg‐Boerstra, Hannah Hunter, Pedro Alves, Enza D'Auria, Anna Comotti, Liam O'Mahony, I‐Jen Wang, Corina Bocsan, Isabel Skypala, Carlo Agostoni, Gavriela Feketea, Camelia Elena Berghea, Inês Pádua, Nikolaos G. Papadopoulos, Carina Venter

**Affiliations:** ^1^ Department of Nutritional Sciences and Dietetics, School of Health Sciences International Hellenic University Thessaloniki Greece; ^2^ Pediatric Area Fondazione IRCCS Ca' Granda‐Ospedale Maggiore Policlinico Milan Italy; ^3^ Department of Clinical Sciences and Community Health Università Degli Studi di Milano Milan Italy; ^4^ Department of Life Sciences, School of Life and Health Sciences University of Nicosia Nicosia Cyprus; ^5^ Institute of Child Health, Department of Pediatric Basic Sciences Istanbul University Istanbul Turkey; ^6^ Faculty of Medicine, Department of Pediatrics, Division of Pediatric Allergy and Immunology Istanbul University Istanbul Turkey; ^7^ Department of Medicine KU Leuven Leuven Belgium; ^8^ Department of Paediatrics OLVG Hospital Amsterdam The Netherlands; ^9^ Rijnstate Allergy Centre Rijnstate Hospital Arnhem The Netherlands; ^10^ Vlieg Dieticians Private Practice for Nutrition and Allergy Arnhem The Netherlands; ^11^ Guys & St Thomas NHS Foundation Trust London UK; ^12^ Allergy and Clinical Immunology Unit University Hospital of Coimbra Coimbra Portugal; ^13^ Department of Pediatrics, Vittore Buzzi Children's Hospital University of Milan Milan Italy; ^14^ Department of Biomedical and Clinical Sciences University of Milan Milan Italy; ^15^ Occupational Medicine Unit Fondazione IRCCS Ca' Granda Ospedale Maggiore Policlinico Milan Italy; ^16^ Department of Medicine, School of Microbiology, APC Microbiome Ireland University College Cork Cork Ireland; ^17^ Department of Pediatrics, Taipei Hospital Ministry of Health and Welfare Taipei Taiwan; ^18^ School of Medicine National Yang Ming Chiao Tung University Taipei Taiwan; ^19^ College of Public Health China Medical University Taichung Taiwan; ^20^ Pharmacology, Toxicology and Clinical Pharmacology Department Iuliu Hatieganu University of Medicine and Pharmacy Cluj‐Napoca Romania; ^21^ Royal Brompton & Harefield Hospitals Part of Guys & St Thomas' NHS Foundation Trust London UK; ^22^ Department of Inflammation & Repair Imperial College London UK; ^23^ Pediatric Allergy Outpatient Clinic, Department of Pediatrics ‘Karamandaneio’ Children's Hospital of Patra Patras Greece; ^24^ Department of Pediatrics ‘Carol Davila’ University of Medicine and Pharmacy Bucharest Romania; ^25^ Department of Pediatrics Clinical Emergency Hospital for Children ‘Marie Sklodowska Curie’ Bucharest Romania; ^26^ Department of Sciences University Institute of Health Sciences Gandra Portugal; ^27^ 4HB/UCIBIO – Translational Toxicology Research Laboratory Gandra Portugal; ^28^ Allergy Department, 2nd Pediatric Clinic National and Kapodistrian University of Athens Athens Greece; ^29^ Section of Allergy & Immunology, Department of Pediatrics, Children's Hospital Colorado University of Colorado School of Medicine Aurora Colorado USA; ^30^ Section of Allergy and Clinical Immunology Children's Hospital Colorado Aurora Colorado USA

**Keywords:** allergies, artificial intelligence, diet quality, dietary diversity, digital health application, immune health, personalized nutrition, ultra‐processed foods

To the Editor,

Immune health is strongly shaped by dietary diversity, nutrient quality, and the degree of ultra‐processed food (UPF) intake.^1^ Over recent decades, immune‐mediated diseases including allergies,^2^ autoimmune disorders,^3^ and infectious diseases,^3^ have risen sharply. Evidence consistently demonstrates that diverse, nutrient‐rich, and minimally processed dietary patterns positively modulate immune function by influencing inflammatory processes, microbiota composition, and micronutrient sufficiency.^1^, ^4^ Conversely, diets characterized by high UPF consumption,^5^, ^6^ or nutrient insufficiency contribute to immune dysregulation and increased susceptibility to inflammatory conditions.^1^

Validated dietary indices are reliable predictors of nutrient adequacy, reduced inflammation, and lowered risk of immune dysfunction.^7^ Yet, their practical application in real‐time, individual‐level dietary monitoring remains limited. Digital health tools have proliferated, but most emphasize calorie counting and weight management rather than immune resilience.^8^ Current gaps include the lack of integration of immune‐related outcomes, reliance on closed or non‐transparent food databases, absence of automated dietary diversity scoring aligned with global sustainability guidelines, and poor adaptability to diverse cultural and linguistic contexts.

To address these limitations, we propose the development of ZENIA, a progressive web application (PWA) designed to transform dietary monitoring into a culturally adaptable, AI‐driven tool for immune‐focused nutrition guidance. The name ZENIA represents ζην (from the ancient Greek “to live”), eating, nutrition, immune health, and actions – an integrated framework emphasizing resilience, mindful eating, nutrient sufficiency, and lifestyle balance. This app will combine automated dietary diversity scoring with barcode scanning, food image recognition, and manual entry, all linked to the open‐access OpenFoodFacts database.^8^ By leveraging AI, ZENIA will capture detailed consumption patterns, including not only nutrients but also allergens, UPFs, and food packaging information (ingredients), while linking these to immune‐related symptoms or inflammatory outcomes. This manuscript presents the ZENIA project protocol – currently in the design and early implementation stages – outlining its rationale, planned phases, and methodological framework for future validation.

The current project received support from the European Academy of Allergy and Clinical Immunology (EAACI) through the Task Force “Personalized Nutrition App for Immune Health: Tracking Dietary Habits and Analyzing Immune Outcomes” (Budget code 41116; 2024–2025), for developing the fundamental components of ZENIA. Additionally, support was provided by the Task Force “Ultraprocessed Foods and Allergy Outcomes: Stopping a Global Epidemic” (Budget code 40326; 2024–2025) for conducting the pilot study. As the ZENIA project is in its formative phase, the following sections describe the structured five‐phase development and evaluation plan rather than completed outcomes.

The development will follow a five‐phase model: (1) stakeholder consultation and requirements mapping; (2) app design and AI integration; (3) pilot testing for feasibility and usability; (4) data analysis and evaluation; and (5) optimization and release. Figure 1 illustrates this workflow.

**FIGURE 1:**
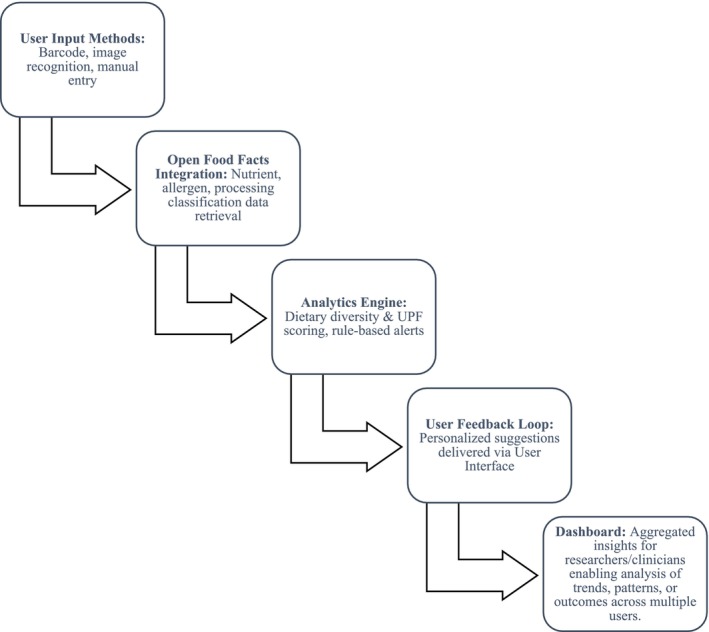
System Architecture Flowchart: ZENIA's technical workflow illustrating data flow and logic processing.

It is a multicenter study involving several participating countries, namely Greece, Italy, Cyprus, the Netherlands, Belgium, the United Kingdom, Portugal, Ireland, Taiwan, Romania, Türkiye, and the United States and is expected to be completed in approximately 24 months.

To map the requirements of the app, a multidisciplinary group – including nutritionists, dietitians, allergists, pediatricians, immunologists, primary care clinicians, behavioral scientists, lifestyle medicine professionals, and app developers have been consulted, with some members also affiliated with stakeholder organizations such as EAACI and European Food Safety Authority (EFSA). Their combined expertise will guide the definition of dietary metrics, including macronutrients, micronutrients, NOVA food processing classification, and allergen presence. They will further define immune‐related outcomes, including self‐reported allergy symptoms, infection frequency, and inflammatory flare‐ups, while also specifying user profile fields such as dietary restrictions, medication use, and pregnancy or breastfeeding status. Immune‐related outcomes will be captured using validated self‐assessment tools, such as Patient‐Oriented Eczema Measure (POEM) for eczema symptoms, Rhinitis Control Assessment Test (RCAT) for rhinitis, and standardized logs for infections. Data will be collected weekly to minimize recall bias, and seasonal variation and medication use will be recorded as covariates.

This process will yield detailed technical and functional specifications for the platform, accompanied by feasibility assessments and compliance checks with the General Data Protection Regulation (GDPR) and other international standards.

The second phase focuses on technical development. The core elements of ZENIA are shown in Table 1. Built as a PWA, the application is accessible across devices. Offline logging and automatic updates maximize usability while eliminating reliance on app store downloads. A core innovation is AI integration into dietary monitoring. Barcode scanning provides instant nutrient and allergen data through the OpenFoodFacts database (https://world.openfoodfacts.org/). OpenFoodFacts offers several advantages: it is open, collaborative, continuously updated, and contains millions of food products worldwide, with detailed information on ingredients, nutrients, allergens, additives, and packaging. Its global coverage supports multicenter projects by allowing standardized coding while reflecting local diets. Furthermore, its open‐access nature facilitates transparency, reproducibility, and interoperability with other health platforms. UPF classification will follow the NOVA framework using a predefined ruleset derived from ingredient parsing. When fields are incomplete or conflicting, manual adjudication will be performed on a gold‐standard subset to estimate inter‐rater reliability and classification error bounds.

**TABLE 1:** Core App Functions – AI Components, Immune Relevance and User Examples.

Function	AI/Digital component	Immune health relevance	User interaction example
Barcode Scanning	Optical scanner connected to Open Food Facts database	Identifies allergens, macronutrient, micronutrient and UPF content influencing immune modulation	“…contains sesame – suggesting alternative whole grain.”
Image Recognition	AI model identifies foods and ingredients from image	Supports accurate dietary diversity tracking	“Detected chickpea salad – diversity score updated.”
Manual Logging	Predictive entry linked to Open Food Facts	Ensures logging completeness	User types “vegetable stew,” app matches to closest item and logs nutrient data.
Dietary Diversity Scoring	Automated based algorithm	Linked to better immune tolerance	“Score 5/9—add leafy greens or legumes to improve.”
Nutritional Guidance	Rule‐based engine (if–else logic)	Promotes variety and nutrient sufficiency and macronutrient balanced diet	“Try adding fermented yogurt for probiotics.” Warning for dificiencies or disbalance in macro nutrients + suggestions?
Allergen Alert	Cross‐check between ingredient data and user profile	Prevents exposure to allergens	“Bread contains egg—avoid if allergic.”
Cultural & Language Adaptation	Localized content for each country	Enhances relevance and engagement among diverse users	Spanish version shows “lentejas” (lentils) recipes.
Future Modules (planned features)	Psychological, Sleep, Activity, Sustainability, Skincare modules	Broader immune‐supportive lifestyle integration	N/A (future enhancement phase)

An AI‐driven image recognition model enables food logging via meal photographs, with automated portion and ingredient estimation. The image‐recognition model will be trained on a balanced dataset representing diverse cuisines. Evaluation will include Top‐1/Top‐5 accuracy, per‐ingredient F1‐score, and portion‐size mean absolute error, stratified by cuisine and country to identify bias. Localization will use forward–back translation and co‐design workshops in each country to ensure culturally appropriate terminology and imagery. All translations will undergo expert review for semantic and functional equivalence.

Manual logging is also supported, enhanced by predictive text linked to the database. Figure 2 presents the Zenia User Journey.

**FIGURE 2:**
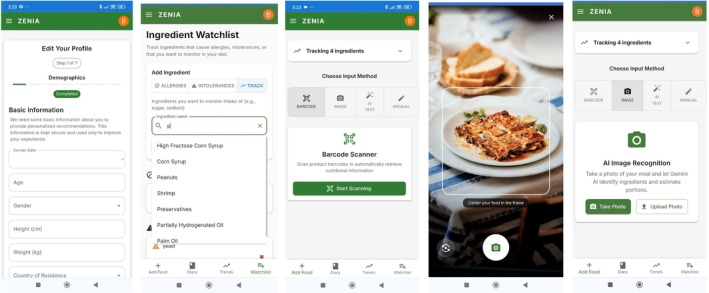
ZENIA User Journey. Screenshots illustrating key user interactions in the prototype version of the ZENIA app. 2.1 and 2.2 Onboarding & Profile Setup. User personalizes their profile (health conditions, dietary restrictions, language preferences). 2.3, 2.4, and 2.5 Dietary Logging Interface. Options: barcode scan, food photo upload (image recognition), manual entry.

To assess dietary adequacy, ZENIA will calculate automated diversity scores in real time. These scores include validated indices such as the WHO dietary diversity indicator.^9^ The system will generate prompts when diversity falls below thresholds. For example, it may suggest legumes for protein variety, leafy greens for vitamin A, or fermented foods for gut health. Personalized guidance emphasizes minimally processed foods, plant‐based proteins, micronutrient‐rich options, and alignment with planetary health diet principles. Cultural and linguistic adaptability is central. ZENIA will feature full content translation and culturally relevant foods in its suggestions and image recognition library, ensuring applicability across populations.

The third phase will evaluate feasibility, usability, and acceptability through pilot testing.

Primary outcomes will include usability (System Usability Scale), adherence to logging, and accuracy of diversity scoring. Secondary outcomes will measure changes in dietary diversity, UPF consumption, and associations with self‐reported immune symptoms. No formal a priori power calculation was conducted. The target sample size aims for approximately 20–30 participants per country to provide stable estimates of usability score and to assess cross‐cultural performance of the app. Units of analysis will be user‐level; missing data will be handled via multiple imputation. Secondary endpoints (dietary diversity, UPF intake, immune symptoms) will be treated as exploratory, with false discovery rate correction for multiple comparisons.

Participants will be recruited across countries for cross‐cultural evaluation, with the sample stratified by health status, socioeconomic background, age, and gender to capture variability. A balanced country distribution will be sought, although recruitment feasibility will also be considered. Ethical approval and GDPR compliance will be secured. Participants will complete baseline surveys on diet, immune health, and medical history before using the app for two weeks. During the trial, users will log foods using AI‐assisted features, receive weekly feedback, and complete end‐of‐study surveys and focus groups. In addition to barcode, image recognition, and manual entry, the pilot will evaluate ZENIA's ability to identify allergens declared in ingredient lists and precautionary allergen labelling (PAL) (e.g., “may contain peanuts”). Allergen detection through OpenFoodFacts will be presented as *decision support*, not diagnostic advice. User‐facing disclaimers will emphasize that the database is crowd‐sourced and may be incomplete or inconsistent across regions.

For pilot testing, allergen presence will be verified via photo‐based OCR and manual quality checking. Performance targets will include sensitivity, specificity, and PPV/NPV per allergen category. Where labeling data are incomplete, users will receive a standardized caution message (“Allergen information not verified—consult packaging”).

This will allow preliminary testing of one of ZENIA's most innovative functions while maintaining focus on real‐world usability. Full‐scale validation of allergen detection will follow later phases, but the pilot will provide feasibility and accuracy data for optimization.

The fourth phase will analyze pilot results. Quantitative methods will include descriptive and inferential statistics examining dietary patterns, adherence, and correlations with immune outcomes. Qualitative analysis, based on thematic coding of user feedback, will identify strengths, weaknesses, and opportunities for improvement. Technical evaluation will assess error rates, response times, and offline performance to ensure reliability.

ZENIA is classified as a digital wellness and educational tool providing non‐diagnostic guidance. It is not a medical device. A post‐market monitoring framework will track user‐reported incidents and AI errors, ensuring transparency and continuous improvement. The final phase will integrate pilot feedback to optimize ZENIA's interface, algorithms, and guidance systems. Once refined, the app will be prepared for deployment across platforms, supported by resources to promote engagement and adherence. Ethics approval will be obtained prior to testing, and user data will remain encrypted and securely stored in compliance with GDPR and international standards.

The project will yield a validated dietary monitoring application that leverages AI to enhance dietary tracking and analysis. Pilot evidence will explore links between diet quality, diversity, UPF intake, and immune outcomes. ZENIA will provide a scalable, culturally adaptable tool with applications in clinical and public health contexts.

Beyond its initial focus, ZENIA is being designed for long‐term scalability. Future iterations will expand into lifestyle domains, including psychological well‐being, sleep, physical activity, and sustainability (e.g., reducing food waste, seasonal foods, plant‐forward meals). These modules will support holistic approaches to immune health by addressing modifiable lifestyle factors. For instance, users may track sleep with personalized routines or monitor outdoor activity with GPS‐based prompts to encourage green space exposure. Eco‐conscious eating and maternal or infant skincare modules will further extend the app's relevance.

ZENIA will represent one of the first PWA to integrate open food data, AI‐powered monitoring, and immune‐focused analytics in a culturally adaptable, globally scalable format. By addressing gaps in dietary assessment – particularly immune outcomes, transparent databases, and real‐time diversity scoring – ZENIA will offer a transformative platform for nutrition and immune health. While ZENIA integrates allergen alerts to support decision‐making, it does not replace professional or manufacturer information. It is expected to generate actionable insights for individuals, clinicians, and policymakers, advancing both personalized nutrition and public health.

## AUTHOR CONTRIBUTIONS

**Emilia Vassilopoulou:** Conceptualization; methodology; funding acquisition; investigation; visualization; project administration; writing – original draft. **Beniam Burje:** Methodology; investigation. **Gregorio Paolo Milani:** Methodology; writing – review and editing. **Cevdet Ozdemir:** Methodology; writing – review and editing. **Rosan Meyer:** Methodology; writing – review and editing. **Berber Vlieg‐Boerstra:** Methodology; writing – review and editing. **Hannah Hunter:** Methodology; writing – review and editing. **Pedro Alves:** Methodology; writing – review and editing. **Enza D'Auria:** Methodology; writing – review and editing. **Anna Comotti:** Methodology; writing – review and editing; data curation. **Liam O'Mahony:** Methodology; writing – review and editing. **I‐Jen Wang:** Methodology; writing – review and editing. **Corina Bocsan:** Methodology; writing – review and editing. **Isabel Skypala:** Methodology; writing – review and editing. **Carlo Agostoni:** Methodology; writing – review and editing. **Gavriela Feketea:** Methodology; writing – review and editing. **Camelia Elena Berghea:** Methodology; writing – review and editing. **Inês Pádua:** Methodology; writing – review and editing. **Nikolaos G. Papadopoulos:** Supervision; writing – review and editing; methodology. **Carina Venter:** Methodology; writing – review and editing; supervision.

## FUNDING INFORMATION

This Task Force report was supported by the European Academy of Allergy and Clinical Immunology (EAACI) under the EAACI Task Force “Personalized Nutrition App for Immune Health: Tracking Dietary Habits and Analyzing Immune Outcomes” (Budget code 41116; years 2024–2025) (Allied Health and Primary Care Section) and for the pilot study from the project “Ultraprocessed foods and allergy outcomes: Stopping a global epidemic” (Budget code 40326; years 2024–2025) (Immunomodulation and Nutrition Working Group).

## CONFLICT OF INTEREST STATEMENT

GPM received grants from Angelini S.P.A. and Reckitt Benckiser Healthcare S.P.A. and acted as advisor for scientific projects funded by Angelini S.P.A.; CIB has received honoraria for lectures from AstraZeneca, MagnaPharm, and Stallergenes; ED received consulting fees from Nestlé and speaker fees from Sanofi; P.B.A. reports personal fees from AstraZeneca and GSK; LO received research grants from Chiesi, Reckitt, and Fonterra; BVB received research funding from OLVG Research, Nutricia, Ekhaga Foundation, Research Fund Skin Diseases, and ZonMw, and received consulting or speaker fees from Nestlé and Nutricia; CV has provided consultancy or educational material to Abbott Laboratories, Danone, Mead Johnson, Novalac and Nestle Nutrition Institute. The rest of the authors declare no conflicts of interest.
